# WHAT FAMILY CAREGIVERS THINK AND FEEL WHEN PROXY ASSESSING FROM DIFFERENT PERSPECTIVES

**DOI:** 10.1093/geroni/igz038.3540

**Published:** 2019-11-08

**Authors:** Patricia Egan

**Affiliations:** University of Wisconsin-Madison, Madison, Wisconsin, United States

## Abstract

Dementia family caregivers are routinely enlisted as proxy assessors of care recipient quality of life (QOL). Proxy assessment is not ideal because proxy assessments differ systematically from self-assessments and the assessment process can elicit negative affect from family caregivers. Prompting adoption of the care recipient’s perspective can enhance assessment congruence and may improve the emotional experience for assessors. This study explored family caregivers’ cognitive and affective experiences during QOL proxy assessments made from both their own and care recipients’ perspectives. Thirty-six dementia family caregivers were recruited from senior service agencies. Subjects completed the Quality of Life-Alzheimer Disease (QOL-AD), Caregiver Version using standard instructions to assess QOL across thirteen domains of their care recipient’s life without specifying the perspective to be used. Subjects were next asked to repeat the QOL-AD with instructions to adopt the perspective of their care recipient, as they imagined it to be. Subjects were then interviewed about what they thought and felt during each proxy assessment experience. Content analysis indicated that spontaneous perspective shifts and response shifts frequently occurred. Most subjects (91.7%) reported changed thinking for one or more QOL-AD domains when they were prompted to switch perspectives. Over half (61.12%) reported changed affect when switching perspectives and 90.9% of those experiencing changed affect reported affective improvement. Little or no affective change was reported by 38.89%. Findings suggest awareness of perspective can enhance clinical interpretation of proxy assessed QOL and can inform clinical response to dementia family caregivers who experience negative emotions while proxy reporting QOL.

